# 17-AAG inhibits vemurafenib-associated MAP kinase activation and is synergistic with cellular immunotherapy in a murine melanoma model

**DOI:** 10.1371/journal.pone.0191264

**Published:** 2018-02-26

**Authors:** Sandeep S. Joshi, Shunlin Jiang, Emmanual Unni, Stephen R. Goding, Tao Fan, Paul A. Antony, Thomas J. Hornyak

**Affiliations:** 1 Department of Biochemistry and Molecular Biology, University of Maryland School of Medicine, Baltimore, Maryland, United States of America; 2 Dermatology Branch, Center for Cancer Research, NCI, NIH, Bethesda, Maryland, United States of America; 3 Department of Pathology, University of Maryland School of Medicine, Baltimore, Maryland, United States of America; 4 Department of Dermatology, University of Maryland School of Medicine, Baltimore, Maryland, United States of America; 5 Research and Development Service, VA Maryland Health Care System, Baltimore, Maryland, United States of America; Rutgers University, UNITED STATES

## Abstract

Heat shock protein 90 (HSP90) is a molecular chaperone which stabilizes client proteins with important roles in tumor growth. 17-allylamino-17-demethoxygeldanamycin (17-AAG), an inhibitor of HSP90 ATPase activity, occupies the ATP binding site of HSP90 causing a conformational change which destabilizes client proteins and directs them towards proteosomal degradation. Malignant melanomas have active RAF-MEK-ERK signaling which can occur either through an activating mutation in *BRAF* (*BRAF*^*V600E*^) or through activation of signal transduction upstream of BRAF. Prior work showed that 17-AAG inhibits cell growth in BRAF^V600E^ and BRAF wildtype (BRAF^WT^) melanomas, although there were conflicting reports about the dependence of BRAF^V600E^ and BRAF^WT^ upon HSP90 activity for stability. Here, we demonstrate that BRAF^WT^ and CRAF are bound by HSP90 in BRAF^WT^, NRAS mutant melanoma cells. HSP90 inhibition by 17-AAG inhibits ERK signaling and cell growth by destabilizing CRAF but not BRAF^WT^ in the majority of NRAS mutant melanoma cells. The highly-selective BRAF^V600E^ inhibitor, PLX4032 (vemurafenib), inhibits ERK signaling and cell growth in mutant BRAF melanoma cells, but paradoxically enhances signaling in cells with wild-type BRAF. In our study, we examined whether 17-AAG could inhibit PLX4032-enhanced ERK signaling in BRAF^WT^ melanoma cells. As expected, PLX4032 alone enhanced ERK signaling in the BRAF^WT^ melanoma cell lines Mel-Juso, SK-Mel-2, and SK-Mel-30, and inhibited signaling and cell growth in BRAF^V600E^ A375 cells. However, HSP90 inhibition by 17-AAG inhibited PLX4032-enhanced ERK signaling and inhibited cell growth by destabilizing CRAF. Surprisingly, 17-AAG also stimulated melanin production in SK-Mel-30 cells and enhanced TYRP1 and DCT expression without stimulating TYR production in all three BRAF^WT^ cell lines studied as well as in B16F10 mouse melanoma cells. *In vivo*, the combination of 17-AAG and cellular immunotherapy directed against Tyrp1 enhanced the inhibition of tumor growth compared to either therapy alone. Our studies support a role for 17-AAG and HSP90 inhibition in enhancing cellular immunotherapy for melanoma.

## Introduction

Heat shock protein 90 (HSP90) is a molecular chaperone which stabilizes the activity of overexpressed and/or mutated signaling proteins commonly found in cancer. The stabilizing properties of HSP90 on client proteins have, in part, been discerned through an analysis of the effects of specific chemical inhibitors of the HSP90 ATPase activity. These include inhibitors of the benzoquinoid ansamycin class, such as geldanamycin (GA) and its semi-synthetic derivatives, 17-allylamino-17-demethoxygeldanamycin (17-AAG) and 17-dimethylaminoethylamino-17-demethoxygeldanamycin (17-DMAG). ATP binding and hydrolysis in an N-terminal pocket of HSP90 [1] drives a conformational switch from an open configuration, where the client protein is bound initially in a complex with co-chaperones HSP70, HSP40, HOP, and HIP; to a closed state with Cdc37, p23, and immunophilins. In the closed state, HSP90 interacts with client proteins, such as ERBB2 (HER-2) [2], AKT [3], and CRAF (Raf-1) [4, 5] to maintain their conformational stability. Inhibition of the closed state following occupancy of the ATP binding site by GA or one of its analogs causes degradation of the client protein through the proteasomal pathway [6–8].

Activation of the Raf family kinases BRAF and CRAF [9–11] is important for the pathogenesis of malignant melanoma [12, 13]. Activation can occur either through a gain of function somatic mutation in *BRAF*, generating the *BRAF*^*V600E*^ substitution mutation found commonly in melanomas arising on non-chronically sun-exposed skin [9, 14, 15]. Alternatively, activation of wild-type BRAF (BRAF^WT^) and CRAF can occur through activating mutations in *NRAS*, or *KIT*, or and through *NF1* deletion [16], which occur in approximately 20% of melanomas [14]. These findings have prompted investigations of the efficacy of GA derivatives for inhibiting RAS- and RAF-dependent signaling and reducing the tumorigenicity of melanoma cells. It was reported earlier BRAF^V600E^ stability was reduced by 17-AAG in A375 human melanoma cells, whereas BRAF^WT^ in CHL melanoma cells was less affected. However, CRAF was degraded, and phosphorylation of ERK inhibited, in each of these cell lines. Only BRAF^V600E^, but not BRAF^WT^ or CRAF, was associated with HSP90 [17]. In a similar study, sensitivity of BRAF to 17-AAG also extended selectively to melanoma cell lines with BRAF^V600E^ mutations. In 4/4 human melanoma cell lines with BRAF^WT^ (SK-Mel-2, SK-Mel-31, SK-Mel-147, and SK-Mel-103), no degradation occurred with 17-AAG concentrations up to 2.5 μM, whereas 17-AAG induced degradation in 5/5 cell lines with BRAF^V600E^ (or BRAF^V600D^) mutations (SK-Mel-1, SK-Mel-5, SK-Mel-19, SK-Mel-28, and WM 266.4). Nonetheless, 17-AAG inhibited melanoma cell proliferation regardless of BRAF mutational status [18], perhaps due to these cells’ dependence upon CRAF signaling in melanomas with activating *NRAS* mutations [11] as well as activation of CRAF by BRAF^WT^ under these conditions [19]. These data suggest that BRAF^V600E^ in melanoma is an HSP90 client protein whose degradation induced by 17-AAG is potentially very important for its inhibitory effects upon melanoma cell growth. However, stabilization of disease course noted in a metastatic melanoma patient with an *NRAS* activating mutation, but BRAF^WT^, during a phase I clinical trial [20] suggests that the effect of 17-AAG on the *NRAS* mutant subset of melanomas requires further consideration.

In this report, we demonstrate that melanoma cells that either harbor activating *NRAS* mutations with BRAF^WT^ or harbor the BRAF^V600E^ mutation with wild-type *NRAS*, the BRAF is bound by HSP90 and under certain conditions is subject to degradation by HSP90 inhibition with 17-AAG. We confirm that 17-AAG inhibits melanoma cell proliferation with each mutational subtype. We also show that 17-AAG can suppress both cell proliferation and the paradoxical activation of ERK [21, 22] in NRAS melanoma cells treated with BRAF inhibitor N-(3-(5-(4-chlorophenyl)-1H-pyrrolo[2;3-b] pyridine-3-carbonyl)-2,4-difluorophenyl) propane-1- sulfonamide (PLX4032 or vemurafenib). Since one mechanism of resistance to the anti-tumor effects of vemurafenib is the mutational or growth factor-induced activation of RAS [23, 24], concurrent administration of BRAF inhibitors and 17-AAG might conceivably delay the growth of resistant clones of melanoma cells in patients with BRAF^V600E^ melanomas. Delays in the development of resistance or therapeutics synergy with inhibitors of HSP90 and BRAF^V600E^ have been shown in melanoma with the HSP90 inhibitors XL888 and AT13387 [25–27]. Here, in addition, we describe a novel activity of the HSP90 inhibitor 17-AAG which induces melanization in BRAF^WT^ melanoma cells in a tyrosinase-independent manner while increasing expression of accessory melanogenic enzymes and tumor antigens [28, 29] TYRP1 and DCT. Correspondingly, we show that 17-AAG and cellular immunotherapy directed against Tyrp1 are synergistic; revealing a new mechanism whereby 17-AAG can potentiate the effects of an established melanoma therapy. These findings extend the range of client proteins of HSP90 susceptible to BRAF degradation and reveal a novel activity of 17-AAG.

## Results

**Fig 1:**
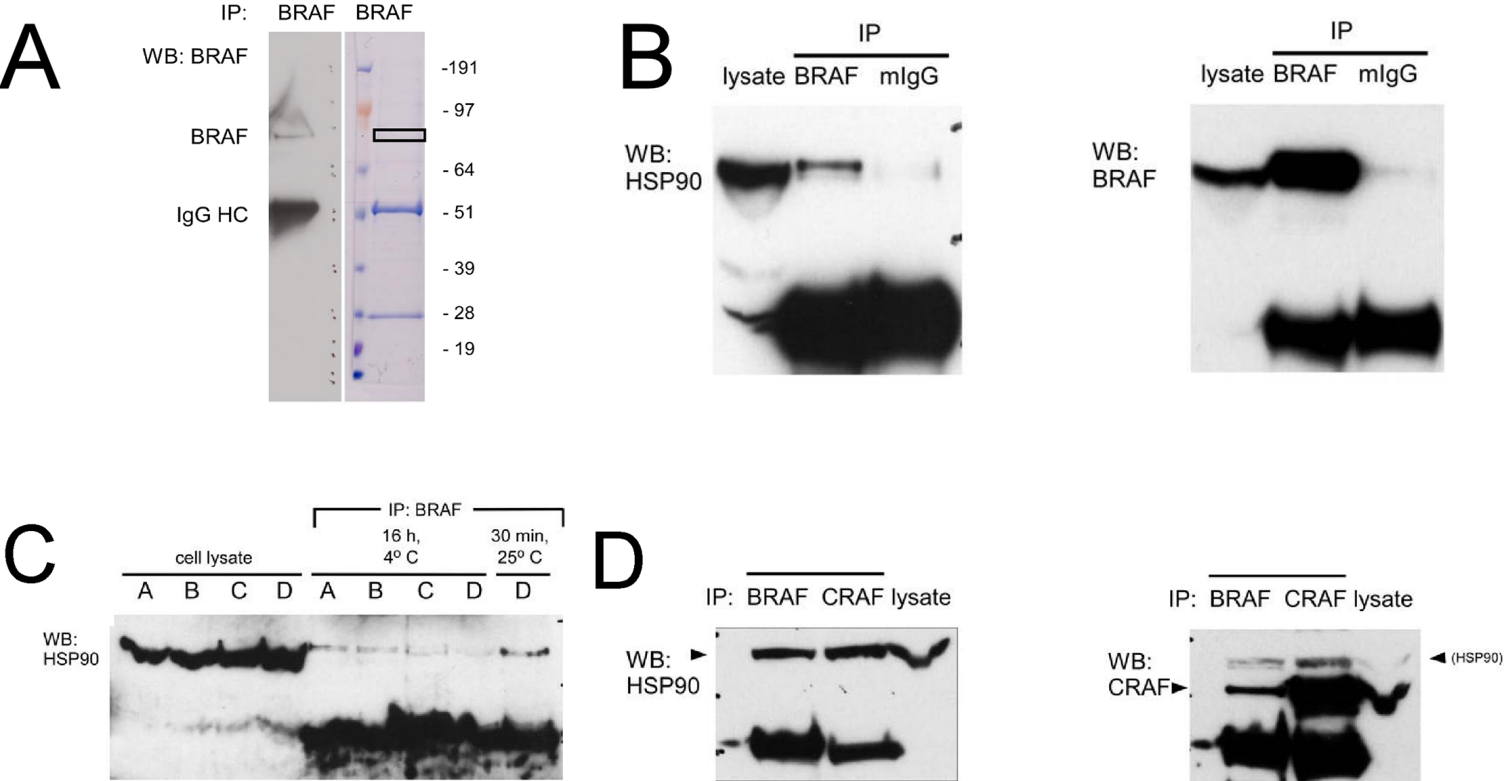
Molecular interactions between BRAF, CRAF, and HSP90 in human melanoma cells. (A) Mass spectroscopic identification of HSP90α and HSP90β as a binding partner of BRAF in human melanoma cells. An immunoprecipitate of BRAF from Mel-Juso melanoma cell lysate was incubated with an anti-BRAF monoclonal antibody, electrophoresed, and subjected to Western blot analysis (left) and Coomassie Blue staining (right). Excision of an ~85 kDa Coomassie-stained band (box) followed by mass spectroscopic analysis revealed peptides corresponding of α- and β-isoforms of HSP90. (B) Co-immunoprecipitation of HSP90 from human melanoma cell lysate with mouse monoclonal anti-BRAF. Following electrophoresis of an anti-BRAF immunoprecipitate, Western blotting (left panel) with monoclonal anti-HSP90 demonstrates HSP90 in the immunoprecipitation complex (BRAF) compared to a control immunoprecipitate with murine IgG (mIgG). (Right panel) Reprobing the membrane in (left panel) with anti-BRAF (right panel) confirms the presence of BRAF in the immunoprecipitate. (C) Immunoprecipitation conditions on stability of HSP90-BRAF interaction. Monoclonal anti-BRAF was incubated with Mel-Juso cell lysate under the following conditions: Condition A, PBS, pH 7.4, 0.1% SDS, 0.5% sodium deoxycholate; Condition B, 50 mM Tris-HCl, pH 7.4, 0.15 M NaCl, 1% NP-40, 0.25% sodium deoxycholate; Condition C, 50 mM Tris-HCl, pH 7.4, 0.1 M NaCl, 1% NP-40 (TENSV) (5); Condition D, 10 mM HEPES, pH 7.35, 20 mM sodium molybdate [31]. (D) (Left panel) Co-immunoprecipitation of HSP90 with either BRAF or CRAF in human melanoma cells. Mel-Juso cell lysate was incubated with either monoclonal anti-BRAF or anti-CRAF. Following electrophoresis and Western transfer, the blot was reprobed with anti-HSP90. (Right panel) Co-immunoprecipitation of CRAF with BRAF. The blot after stripping was reprobed with anti-CRAF. The faint band above the CRAF band is residual signal from previous probing with anti-HSP90.

We were initially interested in characterizing binding partners of the 85 kDa isoform of BRAF in human melanoma cells detected by different anti-BRAF antibodies. Immunoprecipitation of BRAF from Mel-Juso human melanoma cell lysate resulted in the isolation of a strong ~85 kDa band on a Coomassie-stained gel near the area of an immunoreactive Western blot band from an identically-loaded gel lane (Fig 1A). Mass spectroscopic analysis of the excised gel band revealed peptides corresponding to the HSP90-α (HSP 86) and HSP90-β(HSP 84) isoforms of HSP90 (Table 1 and S1 Appendix). This provided initial evidence that BRAF interacts with HSP90 in human melanoma cells.

**Table 1:** 

Rank	Accession Number	ID	Description	MW kDa	Hits	Sequence coverage	pI	pValue
1	P07900	HS9A_Human	Heat shock protein HSP 90-alpha (HSP 86)	85	9	11%	5.0	2e-7
2	P08238	HS9B_Human	Heat shock protein HSP 90-alpha (HSP 86)	83	9	10%	5.0	6e-7

To confirm this interaction, an enhanced HSP90 signal was observed in an anti-BRAF co-immunoprecipitate in comparison to the signal observed with an immunoprecipitate prepared from a non-specific, isotype-matched antibody (Fig 1B). We evaluated a set of conditions utilized previously to evaluate interactions between RAF/Raf kinases and HSP90 and its orthologues. Of these, we found that conditions minimizing incubation time with the antibody at room temperature enhanced detection of the interaction (Fig 1C). As described previously, HSP90 co-immunoprecipated with CRAF in melanoma cell lysate as well. CRAF also co-immunoprecipitates with BRAF (Fig 1D), implying that there is a triad of interactions between BRAF, CRAF, and HSP90 in human melanoma cells. This is in contrast to interactions studied previously in PC12 cells, where BRAF interacts with HSP90 but CRAF does not [30].

Previous work with CRAF has demonstrated that disruption of the interaction between CRAF and HSP90 [31] with the benzoquinone ansamycin geldanamycin leads to destabilization of CRAF [4] and loss of downstream MAP kinase signaling [5]. Similar findings have been described with BRAF^V600E^ in human cells [17, 18]. We were interested in determining whether BRAF^WT^ in melanoma cell lines such as Mel-Juso where we have demonstrated an interaction with HSP90 were destabilized by HSP90 inhibition, since this has not been previously reported. In addition to Mel-Juso, we selected 2 additional cell lines with activating mutations in *NRAS* (SK-Mel-30 and SK-Mel-2) as well as 2 established melanoma cell lines with a *BRAF*^*V600E*^ mutation (A375 and SK-Mel-28) for comparison. SK-Mel-2 melanoma cells have been reported to contain either an *NRAS*^*Q61R*^ [9, 17] or a *KRAS*^*Q61R*^ [11] mutation, but sequencing of the amplified exons 3 of *NRAS* and *KRAS* in the cells used in our experiments confirmed that the mutation is *NRAS*^*Q61R*^, not *KRAS*^*Q61R*^ (S1 Fig). Incubation of these cultured cells with concentrations of 17-AAG up to 1 μM revealed effects on both BRAF and CRAF. CRAF showed evidence of destabilization in 5/5 cell lines (A375, SK-Mel-28, Mel-Juso, SK-Mel-30, and SK-Mel-2), whereas BRAF was degraded in 3/5 cell lines (A375, SK-Mel-28 and SK-Mel-2). Despite the interaction between BRAF^WT^ and HSP90 we demonstrated in Mel-Juso cells, BRAF^WT^ was resistant to degradation by 17-AAG in these cells. However, BRAF^WT^ was degraded by 17-AAG in SK-Mel-2 cells, confirming an observation made previously by one group [17] but not by another where BRAF^WT^ was stable following incubation with 17-AAG [18].

**Fig 2:**
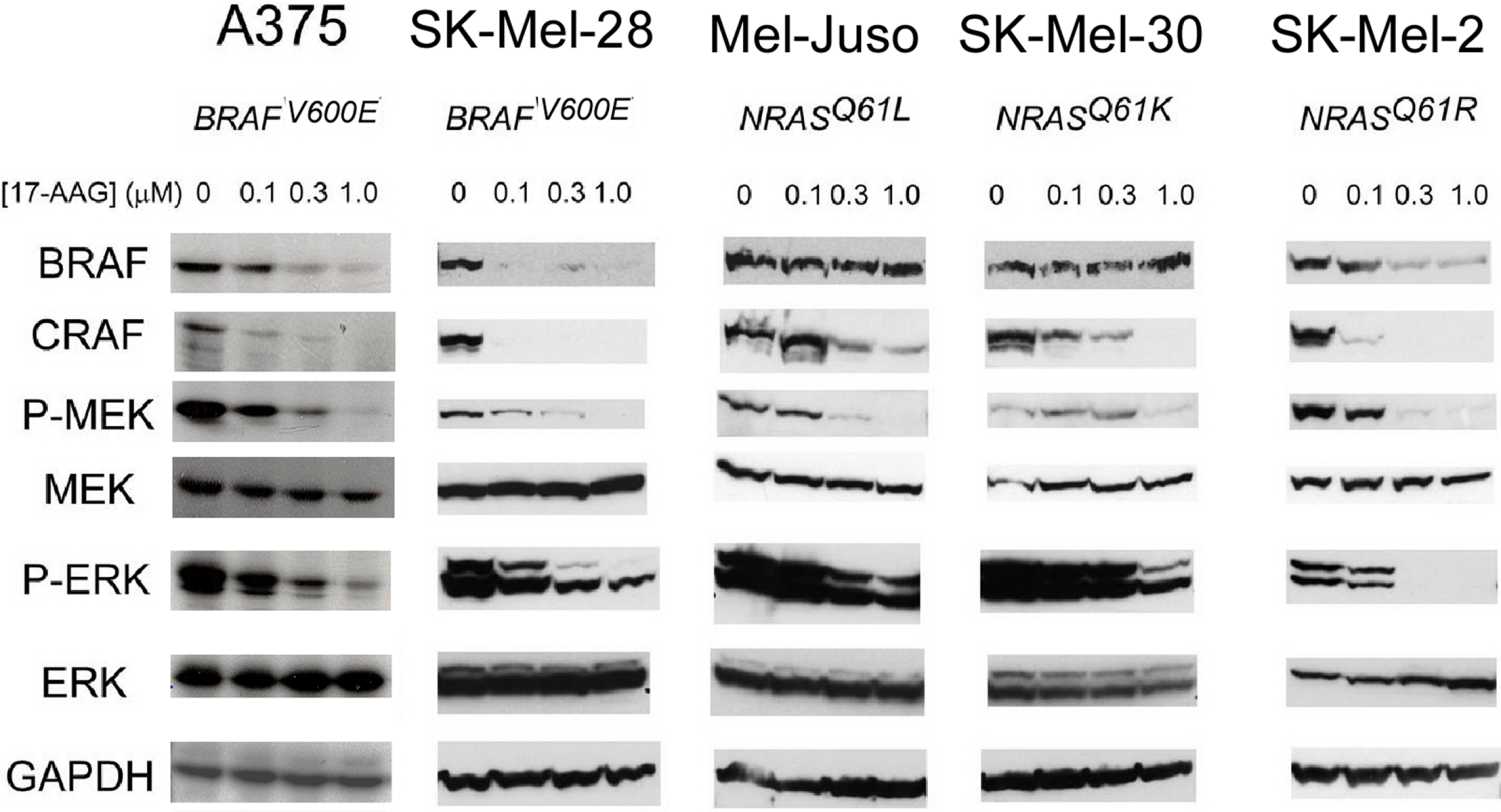
Effects of 17-AAG upon BRAF and CRAF stability and on MAP kinase signaling in human melanoma cells. Human melanoma cells (A375, SK-Mel-28, Mel-Juso, SK-Mel-30, and SK-Mel-2) were incubated with increasing concentrations of 17-AAG (0.1, 0.3, 1.0 μM) for 24 h, and the cell lysates collected were examined for phosphorylation of MAPK pathway by western blot.

We examined how 17-AAG, with consequent degradation of BRAF and CRAF, affected signaling downstream from the RAF kinases. Western blots were examined for relative expression of phosphorylated MEK and ERK kinases. Relative MEK and ERK phosphorylations were diminished at increasing concentrations of 17-AAG in *BRAF*^*V600E*^ and *NRAS* mutated human melanoma cell lines. Inhibition occurs even when BRAF^WT^ (Mel-Juso and SK-MEL-30 cells) was not degraded (Fig 2), confirming the notion that signaling is dependent upon CRAF in this cellular subset [11].

**Fig 3:**
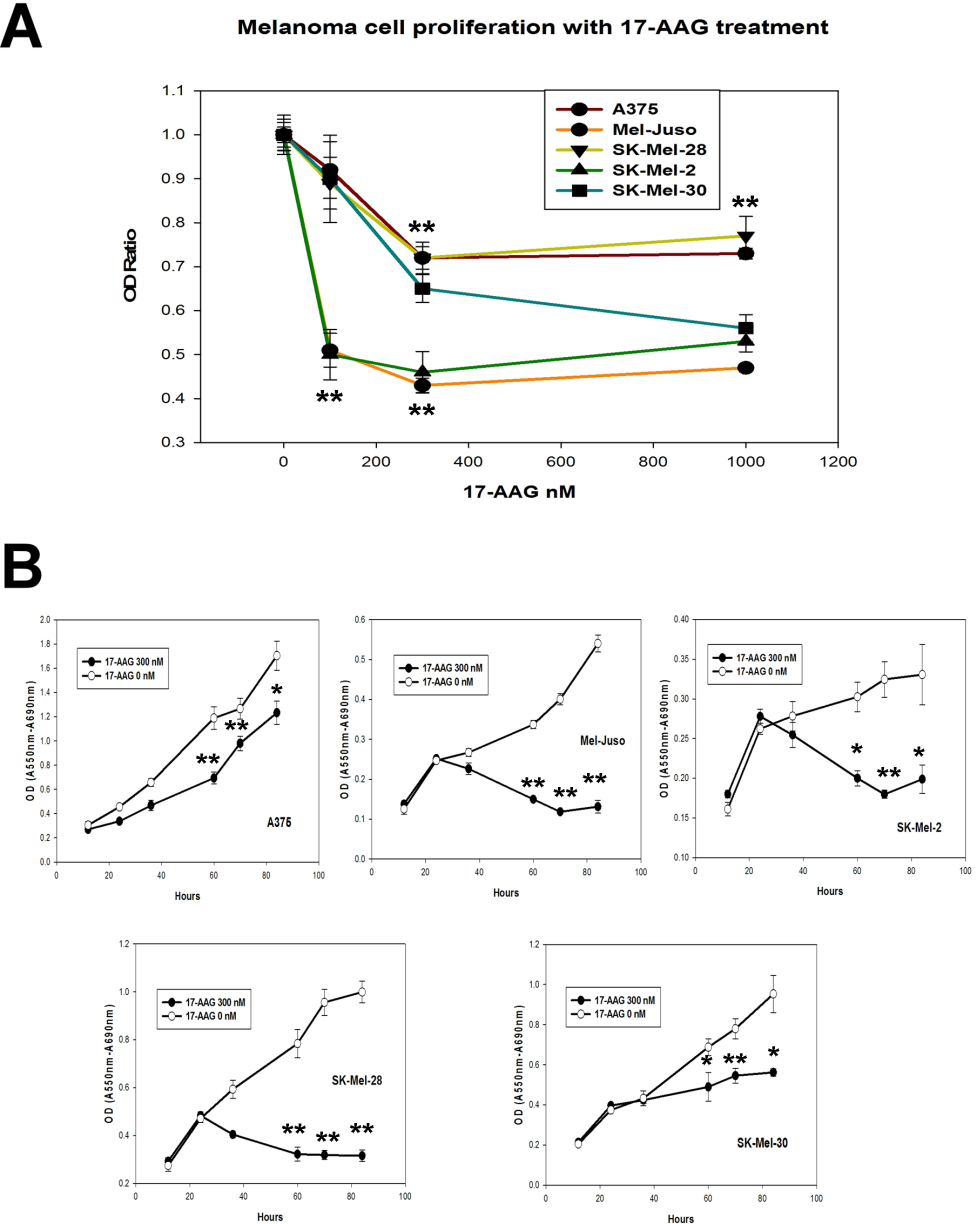
Effect of 17-AAG on cell proliferation in human melanoma cells. (A) Inhibition of melanoma cell viability with increasing concentrations of 17-AAG. Human melanoma cells (A375, SK-Mel-28, Mel-Juso, SK-Mel-30, and SK-Mel-2) were incubated with increasing concentrations (0, 0.1, 0.3, 1.0 μM) of 17-AAG for 48 h. Relative cell number was assessed by differential absorbance at 550 and 690 nm using the MTT assay. (B) Time-dependent growth inhibition of human melanoma cells with 0.3 μM 17-AAG. Human melanoma cells lines were incubated with 0.3 μM 17-AAG for 12, 24, 36, 60, 70, and 84 h before determination of relative cell number using the MTT assay. (**P<0.01; *P<0.05).

To determine whether loss of RAF integrity or inhibition of MAP kinase signaling were correlated with a functional effect of 17-AAG upon cell proliferation, we used the MTT assay to measure the relative rates of cell proliferation of these 5 melanoma cell lines with increasing concentrations of 17-AAG. In a preliminary experiment, cells were incubated with increasing concentrations of 17-AAG (0.1, 0.3, and 1.0 μM). Although partial inhibition of cell viability was observed with 100 nM 17-AAG, for all 5 cell lines, a concentration of 300 nM 17-AAG was sufficient to confer the maximal inhibitory effect (Fig 3A). In a time-dependent manner, 300 nM 17-AAG conferred inhibition of cell proliferation of all 5 melanoma cell lines. With 3/5 cell lines (SK-Mel-2, Mel-Juso, and SK-Mel-28), growth was completely suppressed, whereas with 2/5 cell lines (SK-Mel-30 and A375), growth was partially suppressed at this concentration (Fig 3B).

**Fig 4:**
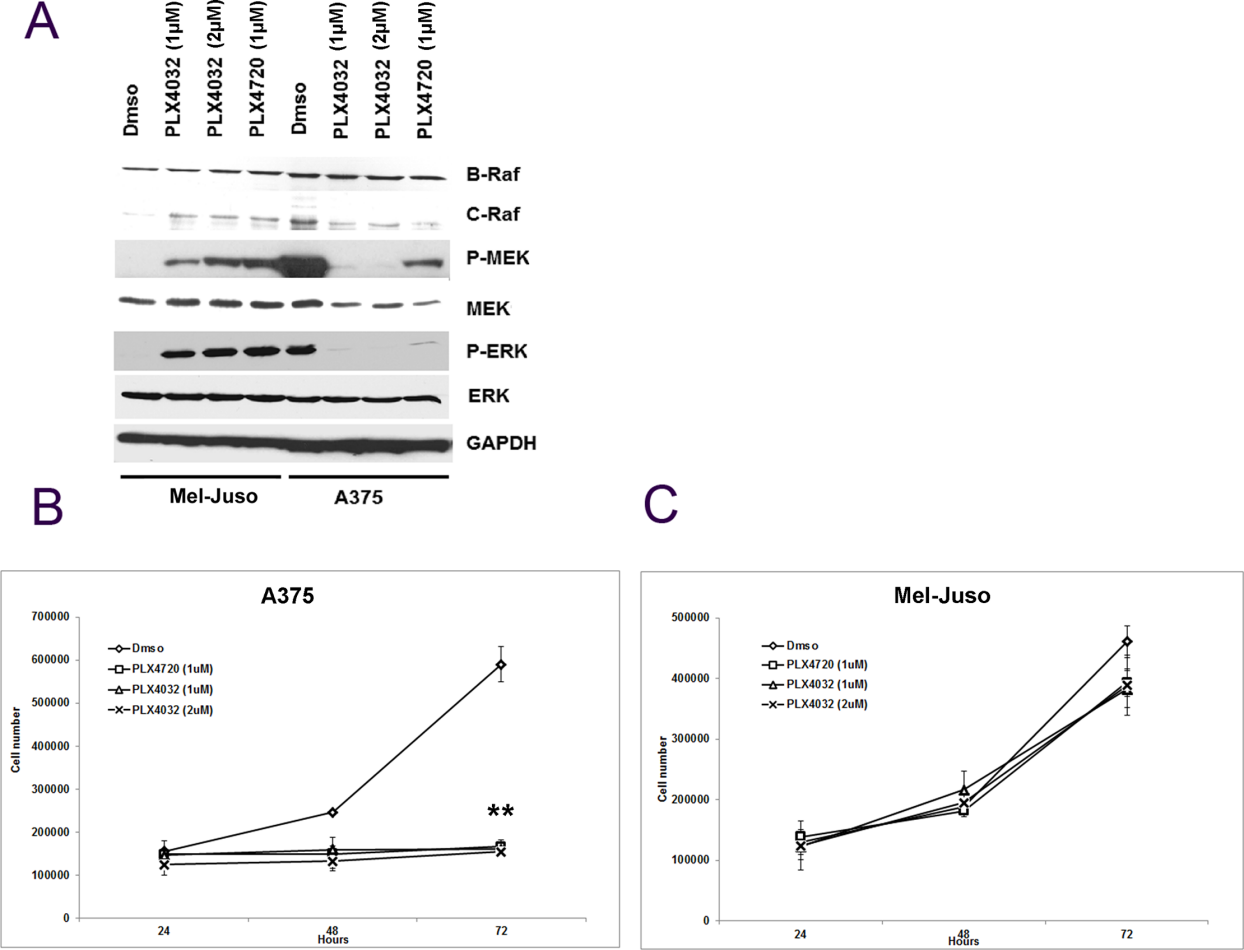
Effect of PLX4032 on MAPK signaling and on proliferation of BRAF^WT^ and BRAF^V600E^ human melanoma cells. (A) Cultured human melanoma cells Mel-Juso and A375 were incubated with PLX4032 (1μM or 2μM) or PLX4720 (1μM) or vehicle (DMSO) for 24h, and cell lysates were studied for protein and phosphoprotein expression by western blot. (B) Human melanoma cell lines Mel-Juso and A375 were incubated with PLX4032 (1μM or 2μM) or PLX4720 (1μM) or vehicle (DMSO) for 24, 48, and 72h. Time-dependent inhibition of PLX4032 and PLX4720 on cell proliferation was determined by counting cells that exclude Trypan blue.

PLX4032, (vemurafenib) an ATP-competitive RAF inhibitor, inhibits ERK signaling and cell proliferation in BRAF^V600E^ melanomas, but enhances the signaling in BRAF^WT^ cells with mutated NRAS [21, 22]. Yet, the ability of 17-AAG to inhibit MAP kinase activity and cell proliferation was retained in NRAS mutant melanoma cell lines (Figs 2 and 3). Therefore we wondered whether 17-AAG could inhibit the PLX4032-induced ERK activation in NRAS mutated human melanoma cells. To achieve this goal, first we examined the effect of PLX4720 and PLX4032 on ERK activation on candidate NRAS mutated (Mel-Juso) and BRAF^V600E^ (A375) cells. As expected, relative MEK phosphorylation and ERK phosphorylation were enhanced in Mel-Juso and inhibited in A375 cells, confirming prior observations [21, 22]. CRAF expression was enhanced in Mel-Juso and slightly diminished in A375 cells, while BRAF^V600E^ and BRAF^WT^ expression was invariant to PLX treatment in each cell lines. PLX4720, a less potent inhibitor of BRAF^V600E^ than PLX4032, showed weaker inhibition of MEK phosphorylation and ERK phosphorylation in A375 cells relative to PLX4032. However, PLX4720 showed similar paradoxical activation of MAPK signaling as indicated by enhanced activation of MEK and ERK phosphorylation similar to PLX4032 in NRAS mutated Mel-Juso cells (Fig 4A). To determine whether altered MAPK signaling was correlated with a functional effect upon cell proliferation, we used Trypan blue exclusion to measure the relative rates of cell proliferation. PLX4720 (1μM) and PLX4032 (1μM and 2μM) suppressed completely the growth of A375 cells (Fig 4B), whereas there was no significant effect on Mel-Juso cells (Fig 4C). Despite the enhanced ERK signaling observed in PLX4032 and PLX4720-treated Mel-Juso cells no additional effect on cell proliferation rate was detected [21]. Since PLX4720 had similar effects on activation of MAPK signaling and cell growth as PLX4032 in BRAF^WT^ melanoma cells, but showed a weaker inhibition of MAPK signaling in BRAF^V600E^ mutant melanoma cells, we decided to used PLX4032, a widely use compound, for further experiments.

**Fig 5:**
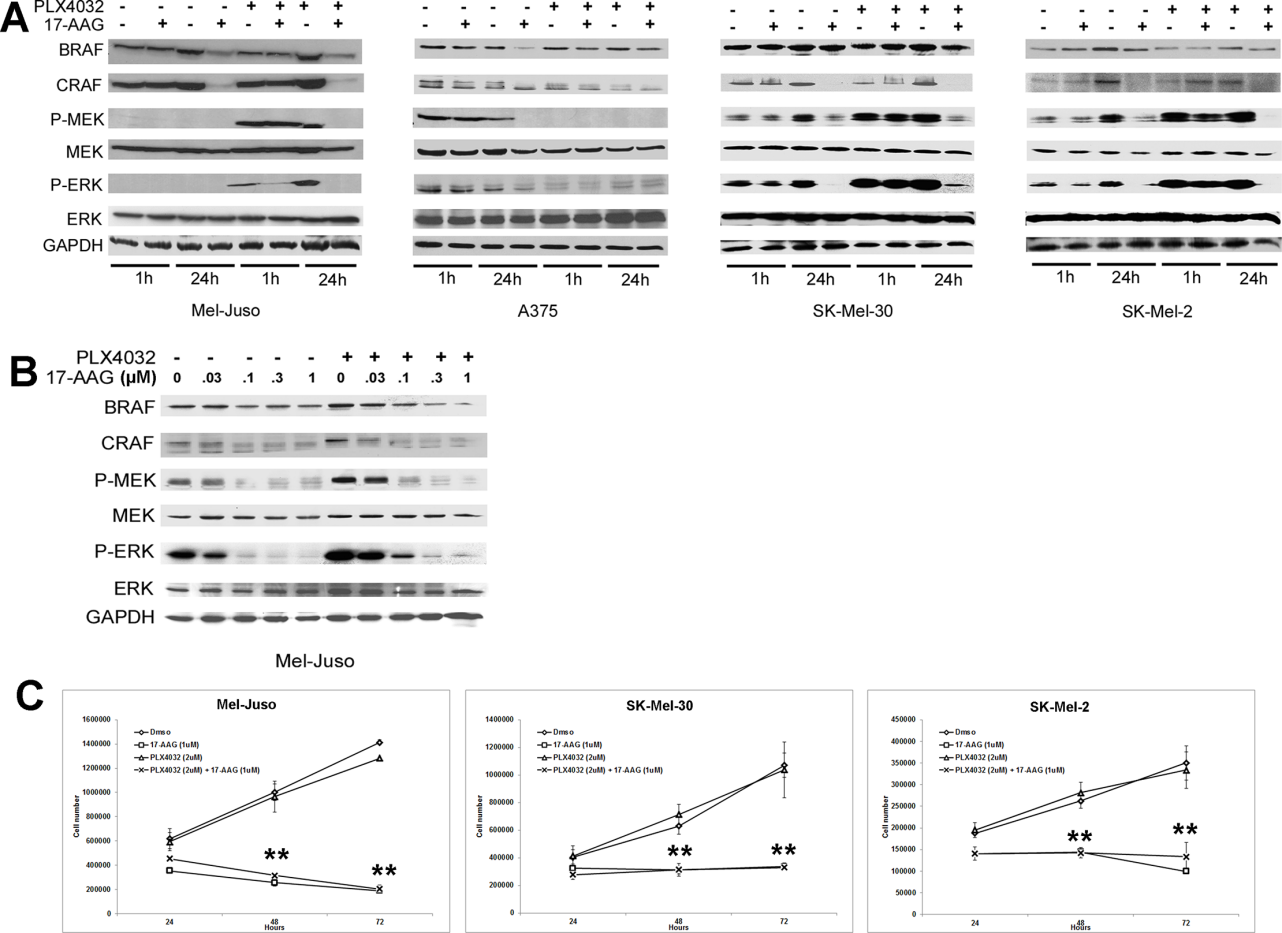
17-AAG treatment inhibits PLX4032 enhanced MAPK signaling in BRAF^WT^ human melanoma cells. (A) Human melanoma cells (Mel-Juso, A375, SK-Mel-30 and SK-Mel-2) were pre-incubated with PLX4032 (2μM) or vehicle (DMSO) for 24h and cells were further incubated with17-AAG (1μM) for 1h or 24h. Cell lysates were examined for the phosphorylation pattern of MAPK pathway by western blot. (B) Cultured human melanoma cell line Mel-Juso was pre-incubated with PLX4032 (2μM) or vehicle (DMSO) for 24h and these cells were subsequently treated with increasing concentrations of 17-AAG (0, 0.03, 0.1, 0.3 or 1μM) for 24h. Cell lysates were collected and studied for phosphoprotein levels of MAPK pathway. (C) Human melanoma cell lines Mel-Juso, SK-Mel-30 and SK-Mel-2 were pre-incubated with PLX4032 (2μM) or vehicle (DMSO) for 24h and were further treated with or without 17-AAG for 24, 48, and 72h. Time-dependent inhibition of human melanoma cell growth with PLX4032 and 17-AAG was determined by counting cells that exclude Trypan blue.

**Fig 6:**
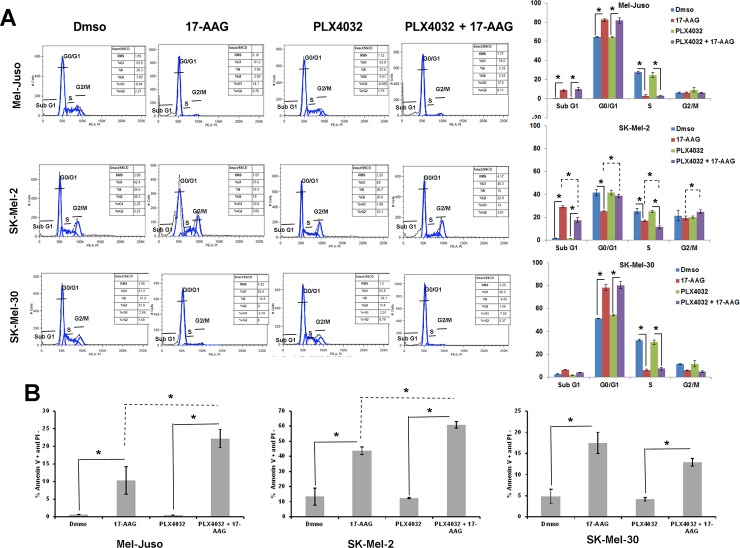
Inhibitory effects of 17-AAG on cell cycle arrest and apoptosis of PLX4032 pretreated human melanoma cells. (A) (left panel) Cell cycle distribution was determined by flow cytometry, on Mel-Juso, SK-Mel-2 and SK-Mel-30 cells pre-incubated with PLX4032 (2μM) or vehicle (DMSO) for 24h with subsequent incubation with or without 17-AAG (1μM) for an additional 72h. Cell cycle profiles were obtained by cell population-based DNA content analysis by flow cytometry (propidium iodide staining) of treated and untreated cells. (Right panel) Graphical representation of the data. The experiment in (left panel) was performed in triplicate and the percent of cells in Sub G1, G0/G1, S, and G2/M was quantified. Data (mean ± SD, n = 3); *P<0.01. (B) Effect of apoptosis was determined by flow cytometry on human melanoma cells (Mel-Juso, SK-Mel-2, SK-Mel-30) pre-incubated with PLX4032 (2μM) or vehicle (DMSO) for 24h, with subsequent incubation with or without 17-AAG (1μM) for an additional 72h. The data is a mean of percentage of Annexin V-positive and PI-negative cell population from three independent experiments. *P<0.01.

These experiments were extended to include 17-AAG. As before, relative MEK and ERK phosphorylation was inhibited in BRAF^V600E^ A375 melanoma cells but enhanced by PLX4032 in all 3/3 BRAF^WT^ melanoma cell lines evaluated (Mel-Juso, SK-Mel-30, SK-Mel-2). However, relative MEK and ERK phosphorylation was inhibited in both BRAF^V600E^ and BRAF^WT^ cell lines when 17-AAG was added after PLX4032. The addition of 17-AAG to PLX4032-treated cells led to a significant degradation of CRAF at 24h in 3/3 BRAF^WT^ cell lines tested (Fig 5A). In addition, to determine the minimum concentration of 17-AAG required to inhibit PLX4032 enhanced ERK signaling, Mel-Juso cells were incubated with increasing concentrations of 17-AAG (.03, 0.1, 0.3, and 1.0μM) with or without PLX4032 pretreatment. MEK and ERK phosphorylation were inhibited to their minimum values by 0.1μM of 17-AAG in control cells but required the higher 0.3μM concentration of 17-AAG in PLX4032 pre-treated cells (Fig 5B). Cell growth studies showed that 2μM PLX4032 by itself conferred no positive or negative effect on cell proliferation in all 3/3 BRAF^WT^ melanoma cell lines. However, 2μM PLX4032 pretreatment in combination with 1μM 17-AAG significantly inhibited cell proliferation over the period of time in 3/3 (Mel-Juso, SK-Mel-30 and SK-Mel-2) BRAF^WT^ cells (Fig 5C). In addition, the cell growth inhibition in PLX4032-pretreated BRAF^WT^ melanoma cells by 17-AAG was further studied for cell cycle inhibition and apoptosis. PLX4032 by itself conferred no positive or negative effect on cell cycle and apoptosis in all 3/3 BRAF^WT^ melanoma cell lines. However, PLX4032-pretreatment in combination with 17-AAG and 17-AAG by itself induced cell cycle arrest in all 3/3 BRAF^WT^ melanoma cell lines. In all three, 17-AAG by itself and in combination with PLX4032 pretreated BRAF^WT^ melanoma cell lines showed highly significant decreases in S phase and increase in G0/G1 arrest. In SK-Mel-2 cells there was significant increase in sub G1 phase in both 17-AAG by itself and in combination with PLX4032-pretreated cells (Fig 6A). In addition, PLX4032 + 17-AAG and 17-AAG alone showed higher Annexin V-positivity in 3/3 BRAF^WT^ melanoma cell lines in comparison to their respective controls. The addition of PLX4032 to 17-AAG also, increased apoptosis in Mel-Juso and SK-Mel-2 cells (Fig 6B). These observations demonstrate that 17-AAG, an Hsp90 inhibitor, can inhibit RAF inhibitor enhanced MAP Kinase signaling through destabilizing CRAF resulting in BRAF^WT^ melanoma cell arrest and apoptosis.

**Fig 7:**
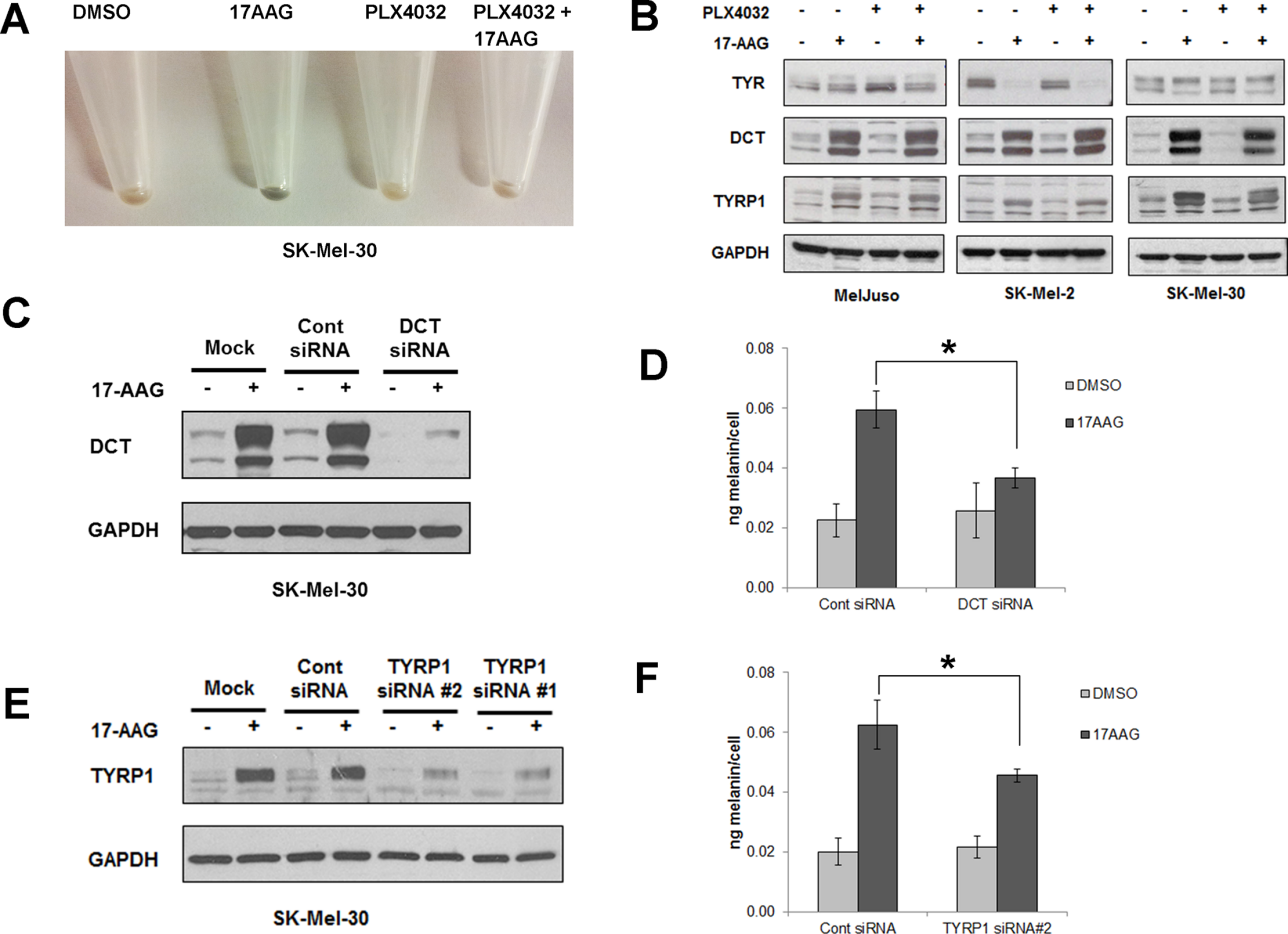
HSP90 inhibition by 17AAG induces pigmentation in human melanoma cells. (A) SK-Mel-30 human melanoma cells pretreated with or without PLX4032 were further treated with 17-AAG or vehicle for 72h, and then harvested and spun down. Data shows the cell pellet color.17-AAG alone induced pigmentation in human melanoma cells. (B) Human melanoma cells (Mel-Juso, A375, SK-Mel-30 and SK-Mel-2) were pre-incubated with PLX4032 (2μM) or vehicle (DMSO) for 24h and subsequently incubated with or without 17-AAG (1μM) for 48h. Cell lysates were collected and studied for the expression of TYR, DCT and TYRP1 proteins by western blot. Data show 17AAG induced DCT and TYRP1 protein expression regardless of PLX4032 treatments whereas TYR was either unchanged (Mel-Juso, SK-Mel-30) or decreased (SK-Mel-2). (C) SK-mel-30 melanoma cells transfected with a control siRNA or DCT siRNA for 24h were incubated with or without 17-AAG for 48h. DCT knockdown was confirmed by western blot. (D) SK-mel-30 melanoma cells transfected with DCT siRNA show significant reduction in melanin content in 17AAG-treated cells in comparison to control siRNA treated cells. Data are represented as percentage of control and SD, measured from three independent experiments. *P < 0.05 vs. control siRNA + 17-AAG. (E) SK-Mel-30 melanoma cells transfected with a control siRNA or 2 different TYRP1 siRNAs (#1, #2) for 24h. They were further treated with or without 17-AAG for 48h. Knockdown of TYRP1 expression was confirmed by collecting cell lysates and running a western blot. (F) SK-Mel-30 melanoma cells transfected with TYRP1 siRNA #2 shows significant reduction in melanin content in 17AAG treated cells in comparison to control siRNA. Data are represented as percentage of control and SD, measured from three independent experiments. *P < 0.05 vs. control siRNA + 17-AAG.

Interestingly, when SK-Mel-30 cells were treated with 17-AAG with or without PLX4032 for 72h, the cells demonstrated a higher visible level of pigmentation (Fig 7A). This observation suggests that 17-AAG, in addition to inhibiting MAPK signaling and human melanoma cell growth, can also stimulate melanin formation. This observation led us to determine whether 17-AAG induced melanin synthesis is correlated with the expression of the canonical melanogenic proteins dopachrome tautomerase (DCT), tyrosinase-related protein-1 (TYRP1), and tyrosinase (TYR). PLX4032 by itself conferred no effect on the expression of DCT, TYRP1 and TYR in all 3/3 BRAF^WT^ melanoma cell lines. However, 17-AAG, in combination with and without PLX4032, increased the expression of DCT and TYRP1, but not TYR, in all 3/3 BRAF^WT^ melanoma cell lines (Fig 7B). It was surprising that the enhanced melanization observed in SK-Mel-30 cells occurred in the absence of an induction of tyrosinase, the rate-limiting enzyme in melanin biosynthesis [32]. To determine whether DCT and/or TYRP1 contribute to 17-AAG-induced melanization, we used siRNAs to deplete these proteins. 17-AAG-induced DCT and TYRP1 induction was significantly blunted in DCT and TYRP1-depleted cells in comparison to control siRNA (Fig 7C and 7E). Depletion of DCT and TYRP1 also reduced total melanin content compared to siRNA-transfected controls (Fig 7D and 7F). Hence 17-AAG stimulates melanization in SK-Mel-30 cells via a tyrosinase-independent mechanism that is dependent upon DCT and TYRP1.

In previous studies, DCT and TYRP1 were identified as melanoma tumor antigens [28, 29]. Their induction by 17-AAG treatment of melanoma cells could render cells more immunogenic against adoptive, cell-based therapy. To test this hypothesis, we utilized a preclinical mouse model whereby the adoptive transfer of naive CD4+ T cells specific for Tyrp1, a melanoma differentiation antigen, results in conversion to cytotoxic T cells *in vivo* and inhibition of tumor growth in lymphopenic murine hosts by direct tumor cell killing [33]. However, murine hosts only exhibited a transient antitumor response, with tumor recurring locally [33, 34]. Hence we asked whether the combination of 17-AAG, responsible for induction of Tyrp1, and adoptive transfer of cytotoxic CD4+ T cells specific for Tyrp1 can enhance the anti-tumor immune response by delaying recurrence of B16 melanomas.

**Fig 8:**
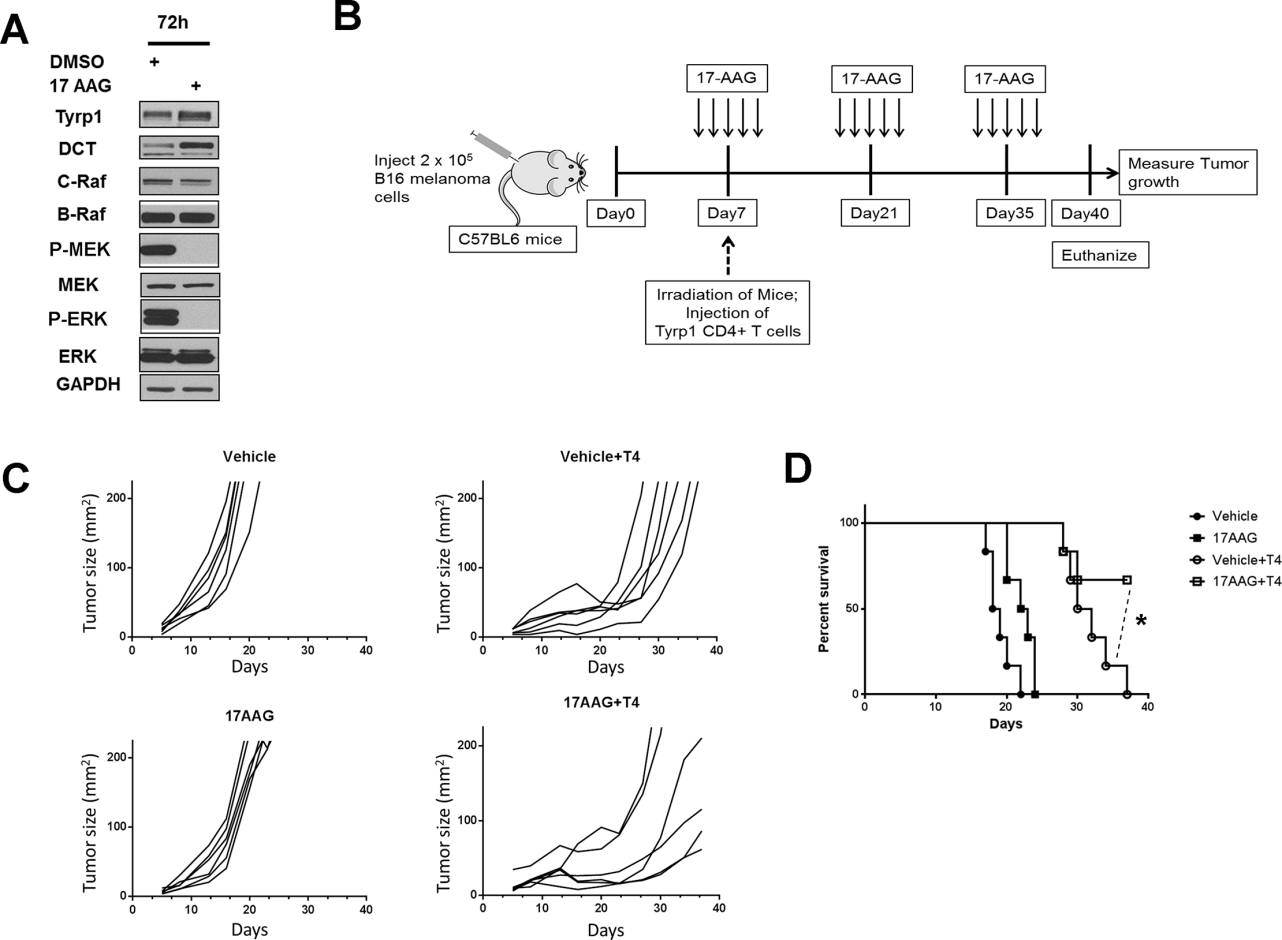
17AAG promotes the inhibition of Tyrp1 specific CD4+ T cell treated melanoma tumor growth. (A) Cultured B16 mouse melanoma cell lines were incubated with 17-AAG (1.0 μM) for 72 h, and cell lysates collected and studied for protein and phosphoprotein expression following SDS-PAGE and Western transfer. (B) The C57BL6 mice were subcutaneously injected with 2 x 10^5^ B16 mouse melanoma cells. Five days after B16 tumor cell injection, the mice received intraperitoneal injection of either 17AAG or vehicle at a dose of 75 mg/Kg body weight x 5 consecutive days. On seventh day after tumor challenge, all mice were irradiated one time with 550 rads (5.5Gy). 50% of 17AAG and 50% of Vehicle treated tumor bearing mice received 2 x 10^5^ numbers of TYRP1-specific CD4+ T cells (T4 cells) were injected intravenously. The 17-AAG dose of 75 mg/kg x 5 consecutive days was repeated every 2 weeks following initial administration of 17-AAG until the end of experiment. (C) The time dependent effect on the B16 melanoma tumor growth in C57BL6 mice treated either with Vehicle, 17AAG, Vehicle+T4 cells or 17AAG+T4 cells were determined by measuring their growth at regular intervals till the end of the experiment (n = 6). Data is a representative of two independent experiments. (D) The survival curve analysis shows that mice receiving no T4 cell transfer and only T4 cell transfer had no surviving mice at day 40. (*P<0.05 is a comparison between Vehicle+T4 cells and 17AAG+T4 cells as indicated by dotted lines).

First, we determined whether HSP90 inhibition by 17-AAG exhibited similar effects upon Tyrp1 and Dct expression in cultured B16 murine melanoma cells. These experiments confirmed that 17-AAG inhibited MAPK signaling shown by absence of phosphorylated MEK and ERK, and induced expression of Dct and Tyrp1 melanogenic proteins (Fig 8A). To determine if 17-AAG treatment can enhance the immune response of adoptively transferred cytotoxic CD4+ T cells against established melanoma tumors, we subcutaneously injected C57BL6 mice with B16.F10 mouse melanoma cells. On the fifth day after xenografting, mice received either 17-AAG or vehicle for 5 days. 7 days after xenografting, all C57BL6 mice were irradiated and 2 x 10^5^ Tyrp1-specific CD4+ T cells were injected intravenously into selected 17-AAG and vehicle-treated tumor-bearing mice (Fig 8B). Tumor measurements were made twice weekly until 40 days after xenografting or until the experimental endpoint was reached. Adoptive transfer of Tyrp1-specific CD4+ T cells by itself caused significant regression of established tumor relative to vehicle treated melanoma bearing mice (Fig 8C, upper panels). However, similar to prior studies [33, 34] we observed recurrence of melanoma tumor in 6/6 mice receiving CD4+ T cells alone (Fig 8C, upper right panel). In mice receiving the combination of 17-AAG and antigen-specific CD4+ T cells, tumor regrowth was significantly delayed, with only 2/6 mice reaching the experimental endpoints (Fig 8C, right bottom panel). The combination of 17-AAG and Tyrp1-specific CD4+ T cells treated mice showed significantly higher survival at Day 40 after tumor challenge when compared with Tyrp1-specific CD4+ T cells alone (P<0.05, Fig 8D).

## Discussion

Previous reports have considered the activity of 17-AAG on the RAF kinases CRAF, and BRAF, in melanoma cells. A study including two human melanoma xenograft lines showed that CRAF was destabilized by the geldanamycin analogs 17-allylamino-17-demethoxygeldanamycin (17-AAG) and 17-dimethylaminoethylamino-17-demethoxy-geldanamycin (17-DMAG) [35], although this effect was variable when another xenograft line was examined [36]. Subsequent studies with cutaneous and uveal melanoma cells [17, 18, 37, 38] have suggested that BRAF^V600E^ is more sensitive to 17-AAG-induced degradation than BRAF^WT^, a suggestion that has been attributed to preferential or exclusive association of BRAF^V600E^ rather than BRAF^WT^ with HSP90. Here we show that BRAF^WT^, at least in the context of activated NRAS in melanoma cells, binds to HSP90 in melanoma cells in a complex with CRAF. Our ability to detect this interaction was enhanced with a shorter incubation time and inclusion of sodium molybdate that were originally described for studying the interaction between HSP90 and CRAF [31]. Altering experimental conditions to resemble these conditions may favor the ability to detect weaker interactions between HSP90 and other client proteins.

Despite the fact that BRAF^WT^ is bound by HSP90 in Mel-Juso cells (Fig 1), it was not degraded by 17-AAG (Fig 2). However, melanoma cell proliferation was inhibited in these and other cell lines (Fig 3) regardless of whether BRAF underwent degradation. In both our study and those reported previously [17, 18], BRAF proteins have been found to be less sensitive to HSP90 inhibition than CRAF. Importantly, cell proliferation and MAP kinase signaling were inhibited in three melanoma cell lines with *NRAS* activating mutations, Mel-Juso, SK-Mel-30, and SK-Mel-2, where BRAF^WT^ was variably insensitive to 17-AAG. These findings support the notion that CRAF is the effector RAF kinase for signaling to MEK in melanoma cells harboring *NRAS* mutations [11].

Currently, no targeted therapy is available to treat advanced BRAF^WT^ melanomas. Treatment of BRAF^V600E^ melanomas invokes resistance in BRAF^V600E^ melanoma cells, with mechanisms of resistance including the development of upstream mutations in *NRAS* or *KRAS* [24]. One possible strategy to delay the clinical onset of resistance is to co-administer drugs that inhibit the ability of initial *NRAS* or *KRAS-*mutant clones to proliferate, as has been suggested previously with 17-AAG and melanoma [39]. Here we show that co-administration of 17-AAG inhibits the paradoxical activation of MAP kinase signaling observed in PLX-treated BRAF^WT^, NRAS-mutant human melanoma cells, providing experimental support for the notion that co-administration may block the expansion of nascent, *RAS-*mutant clones in PLX-treated melanomas. Recent reports with alternative HSP90 inhibitors support this idea [25–27].

17-AAG also induced melanin formation via a tyrosinase-independent mechanism in SK-Mel-30 cells. MEK inhibition has previously been shown to increase melanogenic gene expression, including tyrosinase expression, in immortalized murine melanocytes [40], and tyrosinase gene and protein expression in B16 murine melanoma cells [41]. Here the 17-AAG dependent, tyrosinase-independent induction of melanin formation in human SK-Mel-30 melanoma cells implicates DCT and TYRP1 in facultative melanization, showing that these accessory melanogenic enzymes [42, 43] can modulate pigmentation without changes in tyrosinase. The prior identification of DCT and TYRP1 as melanoma tumor antigens [28, 29] prompted us to investigate whether melanoma cells became more immunogenic following 17-AAG exposure (Fig 8). Our results confirm that this is the case and represent the first demonstration of a synergistic therapeutic effect of an HSP90 inhibitor with cellular immunotherapy in malignant melanoma. Additional work will be required to determine whether this synergistic effect depends upon tumor antigen specificity or on a broad enhancement of tumor inflammation as has been recently suggested [44].

## Materials and methods

### Cell culture conditions

Cells were obtained as frozen stocks from the following sources: NCI-Frederick Cancer DCTD Tumor/Cell Line Repository (SK-Mel-2, SK-Mel-28), ATCC (A375), and DSMZ (SK-Mel-30, Mel-Juso). For experiments measuring interactions between BRAF, CRAF, and HSP90, Mel-Juso human melanoma cells (DSMZ) were cultured in RPMI-1640 (Gibco) with 10% fetal bovine serum (FBS) at 37^o^ C, 5% CO_2_. For experiments examining MAP kinase signaling and the effect of 17-AAG, PLX4032 and PLX4720 on cell proliferation, cells were cultured in RPMI-1640 (Mel-Juso, SK-Mel-2, SK-Mel-28, SK-Mel-30, A375). To compare the effects of 17-AAG on BRAF and CRAF degradation in SK-Mel-2 cells, cells were cultured in RPMI-1640, DMEM [17], or a 50:50 mixture of DMEM/Ham’s F12 medium [18]. B16.F10 (H-2b), hereafter called B16, is a Tyrp1+ spontaneous murine melanoma cell line and maintained in culture conditions as previously described [45].

### Mass spectrometric identification of HSP90 as BRAF interactor

Cell lysate was prepared from Mel-Juso cells cultured as above by lysing cell pellets in RIPA buffer with protease and phosphorylation inhibitors (20 μg/ml aprotinin, 20 μg/ml leupeptin, 1 mM sodium orthovanadate, 2 mM phenylmethylsulfonylfluoride). BRAF was immunoprecipated from cell lysate by incubating 100 μl lysate with 10 μl antibody solution and 20 μl Protein A-agarose (Pierce) in TENSV buffer [5] with the protease and phosphorylation inhibitors described above at room temperature for 30 min with gentle rolling. The antigen-antibody-Protein A-agarose complex was collected by centrifugation at 3000 g for 1 min. The resulting complex was washed twice with 0.5 ml TENSV buffer with inhibitors and loaded onto lanes of a 10% SDS-PAGE gel for electrophoresis. The gel was Coomassie-stained and the indicated band (Fig 1A) visualized, cut with a sharp blade, and submitted for mass-spectroscopic analysis.

### Co-immunoprecipitation of BRAF, CRAF, and HSP90 proteins

Immunoprecipitation of BRAF was performed as described above. The resulting complex was washed twice with TENSV buffer containing inhibitors and loaded onto lanes of a 10% SDS-PAGE gel for electrophoresis. Following electrophoresis, the gel was subjected to Western transfer and incubation with antibodies. Similar procedures were used for immunoprecipitation of HSP90 and CRAF, except that protein G-agarose, instead of protein A-agarose, was used.

### Inhibition of MAP kinase signaling and evaluation of BRAF and CRAF stability by 17-AAG, PLX4032 and PLX4720

Cells (Mel-Juso, SK-Mel-2, SK-Mel-28, SK-Mel-30, A375 and B16) were grown in 10 cm tissue culture plates under the conditions described previously. 17-AAG (Cancer Therapy and Evaluation Program, National Cancer Institute) at a stock concentration of 10 mM in DMSO was added at final concentrations of 0, 100, 300, and 1000 nM for 24 h or PLX4032 or PLX4720 (ChemieTek. Indianapolis, IN) at a concentrations of 1 and 2 μM for 24h or 48h. Cell pellets were lysed in RIPA buffer with protease and phosphorylation inhibitors as above and protein concentration determined using the Bio-Rad protein assay (Bio-Rad Laboratories, #500–0006).Lysates were loaded onto a 10% SDS-PAGE gel, subjected to electrophoresis, and transferred to a PVDF (Invitrogen) membrane for Western blotting. The following primary antibodies were used: monoclonal anti-BRAF (F7, Santa Cruz Biotechnology), polyclonal anti-BRAF (07–453, Upstate/Millipore), monoclonal anti-CRAF (BD Biosciences Pharmingen), monoclonal anti-HSP90 (Stressgen), monoclonal anti-MEK1 (H8, Santa Cruz Biotechnology), polyclonal anti-phospho-Mek1/2 (Ser218/Ser 222, Santa Cruz Biotechnology), polyclonal anti-ERK2 (C-14, Santa Cruz Biotechnology), monoclonal anti-phospho-ERK (E4, Santa Cruz Biotechnology), Dct (α-PEP8), Tyrp1(α-PEP1) and Tyr (α-PEP7) are rabbit polyclonal antibodies, gifts from Dr. Vincent Hearing, NIH. The dilutions used range from 1:200–1:2000. HRP conjugated secondary antibodies were either purchased from Millipore or Santa Cruz Biotechnology and were used at 1:5000 dilutions.

### MTT assay and cell proliferation assay for the effect of 17-AAG, PLX4032 and PLX4720 on human melanoma cells

To assess the effect of 17-AAG on cell viability and proliferation in human melanoma cells, the 3-(4,5-dimethylthiazol-2-yl)-2,5-diphenyltetrazolium bromide (MTT) assay was used by utilizing the Cell Proliferation Kit I (MTT) from Roche Biotechnology. Briefly, melanoma cells (Mel-Juso, SK-Mel-2, SK-Mel-28, SK-Mel-30, A375) were seeded in individual wells of a 96-well plate in hexatuplicate with 200 μl of the indicated cell culture medium and incubated for 24 h. The initial medium was removed, and replaced with 100 μl of fresh medium with increasing concentrations (0, 100, 300, or 1000 nM) of 17-AAG and incubated for 0–96 hours. Following the incubation period, 10 μl of MTT labeling reagent was added to each well and incubated at 37^o^ C for an additional 4 hours. 100 μl of solubilization reagent was added to each well and the colorimetric reaction was allowed to develop overnight in the 37^o^ C incubator. An ELISA plate reader was used to record the optical densities at 550 nm and 690 nm.

To assess the effect of PLX4032 and PLX4720 on human melanoma cell proliferation, the Trypan blue exclusion method was used. Melanoma cells (Mel-Juso and A375) were seeded overnight in individual wells of 6 well plate with 2ml of the indicated cell culture medium. The initial medium was replaced with 2ml of medium containing 1μM of PLX4720, 1 or 2 μM PLX4032 or vehicle for 24, 48, and 72 hours. Time-dependent inhibition of human melanoma cells with PLX was determined by counting Trypan blue-excluded cells.

To determine the effect of 17-AAG on PLX resistant WT BRAF melanoma cells, trypan blue exclusion method was used. Melanoma cells (Mel-Juso, SK-Mel30, SK-Mel2) were treated with 2 μM PLX4032 or vehicle for 24h as described above and were further treated with or without 17-AAG for 24, 48 and 72 hours. Time-dependent inhibition of human melanoma cells with PLX4032 and 17-AAG was determined by counting Trypan blue-excluded cells.

### Flow cytometry assay

To determine the effect of 17-AAG on cell cycle arrest of PLX-resistant WT BRAF melanoma cells (Mel-Juso, SK-Mel-30, SK-Mel-2), cells were pre-incubated with PLX4032 (2μM) or vehicle (DMSO) for 24h and these cells were incubated subsequently with or without 17-AAG (1μM) for an additional 72h. Post treatments, cells were fixed in chilled ethanol (70%), washed with PBS, and then treated with DNase-free RNase (200 μg/ml) for 1 hour at 37°C, stained with PI (40 μg/ml) and kept in the dark for 15 minutes at 20–25°C. Staining was measured on a FACSCanto II flow cytometer (BD Biosciences, San Jose, CA), and percentages of cells in different cell cycle phases were determined using FlowJo Software (Tree Star, Inc., Ashland, OR).

To determine the effect of 17-AAG on apoptosis of PLX-resistant WT BRAF melanoma cells (Mel-Juso, SK-Mel-30, SK-Mel-2), cells were pre-incubated with PLX4032 (2μM) or vehicle (DMSO) for 24h and these cells were subsequently incubated with or without 17-AAG (1μM) for an additional 72h. Post treatments, cells (2–3 × 10^5^) were washed with PBS, resuspended in annexin V binding buffer (1x), stained with annexin V-FITC (1 μL) and PI (2 μL) incubated at room temperature in the dark, then washed and acquired on a BD LSRII flow cytometer (BD Biosciences, San Jose, CA) and analyzed with FlowJo Software (Tree Star, Inc., Ashland, OR).

### siRNA inhibition of DCT and TYRP1

SK-Mel-30 cells seeded into 6 well plates at 2.0 × 10^5^ were treated with either 40 nM DCT siRNA or TYRP1 siRNA #1 or #2 or 40 nM control non-targeting siRNA (Customized at Integrated DNA Technologies, Inc.) using the Lipofectamine RNAiMax transfection reagent (Invitrogen) as per the manufacturer's instructions. DCT and TYRP1 inhibition was confirmed by western blotting 120 h after transfection. There was significant decrease in DCT and TYRP1 protein relative to control siRNA treated SK-Mel-30 cells at the time point.

The customized DCT and TYRP1 siRNA sequences are as follows.

DCT siRNA

Sense strand: 5’ rGrCrArUrGrArCrGrGrUrGrGrArCrArGrCrCrUrArGrUrGAA

Antisense strand: 5’ rUrUrCrArCrUrArGrGrCrUrGrUrCrCrArCrCrGrUrCrArUrGrCrArG

TYRP1 siRNA #1

Sense strand: 5’ rGrArArArCrArCrArGrUrGrGrArArGrGrUrUrArCrArGrUGA

Antisense strand: 5’ rUrCrArCrUrGrUrArArCrCrUrUrCrCrArCrUrGrUrGrUrUrUrCrGrG

TYRP1 siRNA #2

Sense strand: 5’ rGrArArCrUrArCrCrArUrGrCrUrUrUrGrUrUrUrArCrGrUGT

Antisense strand: 5’ rArCrArCrGrUrArArArCrArArArGrCrArUrGrGrUrArGrUrUrCrArC

### Quantification of the melanin content

The cultured cells at the end of experiment were detached from the culture plate using trypsin-EDTA solution and counted in a hemocytometer. Cell suspensions equivalent to 200,000 cells were pelleted and lysed in 1ml of 1N NaOH and the melanin concentration is determined by measuring optical density at 475 nm and compared with a standard curve obtained using melanin isolated from sepia (Sigma). Data of melanin concentration is represented as melanin produced by each cell in a given time [46].

### Mice and adoptive transfer of T cells

C57BL/6 mice were purchased from Veterinary Resources, Program in Comparative Medicine, University of Maryland School of Medicine. Tyrp1 TCR x tyrp-1^bw^Rag1^-/-^ transgenic mice express a transgenic TCR that recognizes the mtyrp1 (113–127) peptide in the context of MHC class II. These mice also are homozygous for the white-based brown mutation, Bw that has a defect in exon 1 of tyrosinase-related protein-1 gene [33, 47].

The C57BL6 mice were injected subcutaneously in the abdomen with 2 x 10^5^ B16 mouse melanoma cells with a 27-gauge needle. This dose is well above the dose required to establish tumors in mice. Tumors were allowed to become established and sizeable for injections. Animals were euthanized at signs of distress and/or cachexia and tumor sizes beyond 200 mm^2^. Five days after B16 tumor cell injection, the mice received intraperitoneal injections of either 17-AAG or vehicle at a dose of 75 mg/Kg body weight x 5 consecutive days [48]. Seven days after tumor challenge, all mice were irradiated one time with 550 rads (5.5Gy). Nine to twelve days after tumor challenge, 2 x 10^5^ Tyrp1-specific CD4+ T cells were injected intravenously into 50% of 17-AAG and 50% of vehicle treated tumor bearing mice. The 17-AAG dose of 75 mg/kg x 5 consecutive days were repeated every 2 weeks following initial administration of 17-AAG until the mice were euthanized. Tumors were measured with digital calipers every 3 to 4 days. The perpendicular diameters were determined and multiplied to generate the area (in mm^2^), as described previously [33].

Tyrp1 CD4+ T cells were sorted from spleens of donor Tyrp1 TCR x Tyrp1^bw^RAG-1^-/-^ as described (Goding SR et al. 2013 J immuno). Spleens were harvested and made into single-cell suspensions. Cells were made devoid of red blood cells by ACK (Ammonium-Chloride-Potassium) lysis. Subsequently, cells were counted and enriched for CD4+ T cells by magnetic bead sorting using a CD4+ T cell enrichment kit (Miltenyl Biotech) as per manufactures instruction. Enriched Tyrp1 CD4+ T cells were counted and resuspended in PBS and used in adoptive transfer studies (2 x 10^5^ cells/mouse).

### Statistical analysis

All statistical analyses were performed using Graphpad Prism 6.0 software. One-way ANOVA was used to analyze data in experiments such as MTT assay, cell proliferation assay, apoptosis assay and quantification of melanin content. Two-way ANOVA was used to analyze data in cell cycle experiments. Survival curves were compared with a log-rank test. *P* values of 0.05 or less were considered significant.

## Supporting information

S1 Appendix Mass spectroscopic analysis of the excised gel band of BRAF revealed peptides corresponding to isoforms of HSP90. Mass spectroscopic analysis of the excised gel band of BRAF from MelJuso melanoma cells showed 9 peptides corresponding to each α- and β-isoforms of HSP90. Nine peptides corresponding to full length sequences of α- and β-isoforms of HSP90 are highlighted in yellow.
(DOCX)

S1 Fig Sequence analysis of *NRAS* and *KRAS* in SK-Mel-2 cells. The human melanoma cell line SK-Mel-2 was screened for the *NRAS*^*Q61R*^ or *KRAS*^*Q61R*^ mutation. The sequencing of the amplified exon 3 of *NRAS* and *KRAS* in the SK-Mel-2 cells showed the mutation is *NRAS*^*Q61R*^ (top), and not *KRAS*^*Q61R*^ (bottom).
(TIF)
