# Supplementary material for: 17-AAG inhibits vemurafenib-associated MAP kinase activation and is synergistic with cellular immunotherapy in a murine melanoma model
Source: PLoS One. 2018 Feb 26;13(2):e0191264. doi: 10.1371/journal.pone.0191264 (PMC5826531; doi:10.1371/journal.pone.0191264)
Supplement: S1 Appendix — Mass spectroscopic analysis of the excised gel band of BRAF from MelJuso melanoma cells showed 9 peptides corresponding to each α- and β-isoforms of HSP90. Nine peptides corresponding to full length sequences of α- and β-isoforms of HSP90 are highlighted in yellow. (DOCX) [file pone.0191264.s001.docx]

**S1 Appendix. Mass spectroscopic analysis of the excised gel band of BRAF revealed peptides corresponding to isoforms of HSP90.**

1) P07900 HS9A_HUMAN

**Heat Shock protein HSP 90-alpha (HSP 86).**

Swiss-prot : Homo sapiens : 9606

1 mpeetqtqdq pmeeeevetf afqaeiaqlm sliintfysn keiflrelis nssdaldkir

61 yesltdpskl dsgkelhinl ipnkqdrtlt ivdtgigmtk adlinnlgti aksgtkafme

121 alqagadism igqfgvgfys aylvaekvtv itkhnddeqy awessaggsf tvrtdtgepm

181 grgtkvilhl kedqteylee rrikeivkkh sqfigypitl fvekerdkev sddeaeeked

241 keeekekeek esedkpeied vgsdeeeekk dgdkkkkkki kekyidqeel nktkpiwtrn

301 pdditneeyg efyksltndw edhlavkhfs vegqlefral lfvprrapfd lfenrkkknn

361 iklyvrrvfi mdnceelipe ylnfirgvvd sedlplnisr emlqqskilk virknlvkkc

421 lelftelaed kenykkfyeq fskniklgih edsqnrkkls ellryytsas gdemvslkdy

481 ctrmkenqkh iyyitgetkd qvansafver lrkhgleviy miepideycv qqlkefegkt

541 lvsvtkegle lpedeeekkk qeekktkfen lckimkdile kkvekvvvsn rlvtspcciv

601 tstygwtanm erimkaqalr dnstmgymaa kkhleinpdh siietlrqka eadkndksvk

661 dlvillyeta llssgfsled pqthanriyr miklglgide ddptaddtsa avteempple

721 gdddtsrmee vd

2) P08238 HS9B_HUMAN

**Heat Shock protein HSP 90-beta (HSP 84).**

Swiss-prot : Homo sapiens : 9606

1 mpeevhhgee evetfafqae iaqlmsliin tfysnkeifl relisnasda ldkiryeslt

61 dpskldsgke lkidiipnpq ertltlvdtg igmtkadlin nlgtiaksgt kafmealqag

121 adismigqfg vgfysaylva ekvvvitkhn ddeqyawess aggsftvrad hgepigrgtk

181 vilhlkedqt eyleerrvke vvkkhsqfig ypitlyleke rekeisddea eeekgekeee

241 dkddeekpki edvgsdeedd sgkdkkkktk kikekyidqe elnktkpiwt rnpdditqee

301 ygefyksltn dwedhlavkh fsvegqlefr allfiprrap fdlfenkkkk nniklyvrrv

361 fimdscdeli peylnfirgv vdsedlplni sremlqqski lkvirknivk kclelfsela

***** 421 edkenykkfy eafsknlklg ihedstnrrr lsellryhts qsgdemtsls eyvsrmketq

481 ksiyyitges keqvansafv ervrkrgfev vymtepidey cvqqlkefdg kslvsvtkeg

541 lelpedeeek kkmeeskakf enlcklmkei ldkkvekvti snrlvsspcc ivtstygwta

601 nmerimkaqa lrdnstmgym makkhleinp dhpivetlrq kaeadkndka vkdlvvllfe

661 tallssgfsl edpqthsnri yrmiklglgi dedevaaeep naavpdeipp legdedasrm

721 eevd

***** Two peptide sequences were found (1) 438 to 447 and (2) 438 to 448.
