# Supplementary figures and images for: 17-AAG inhibits vemurafenib-associated MAP kinase activation and is synergistic with cellular immunotherapy in a murine melanoma model
Source: PLoS One. 2018 Feb 26;13(2):e0191264. doi: 10.1371/journal.pone.0191264 (PMC5826531; doi:10.1371/journal.pone.0191264)

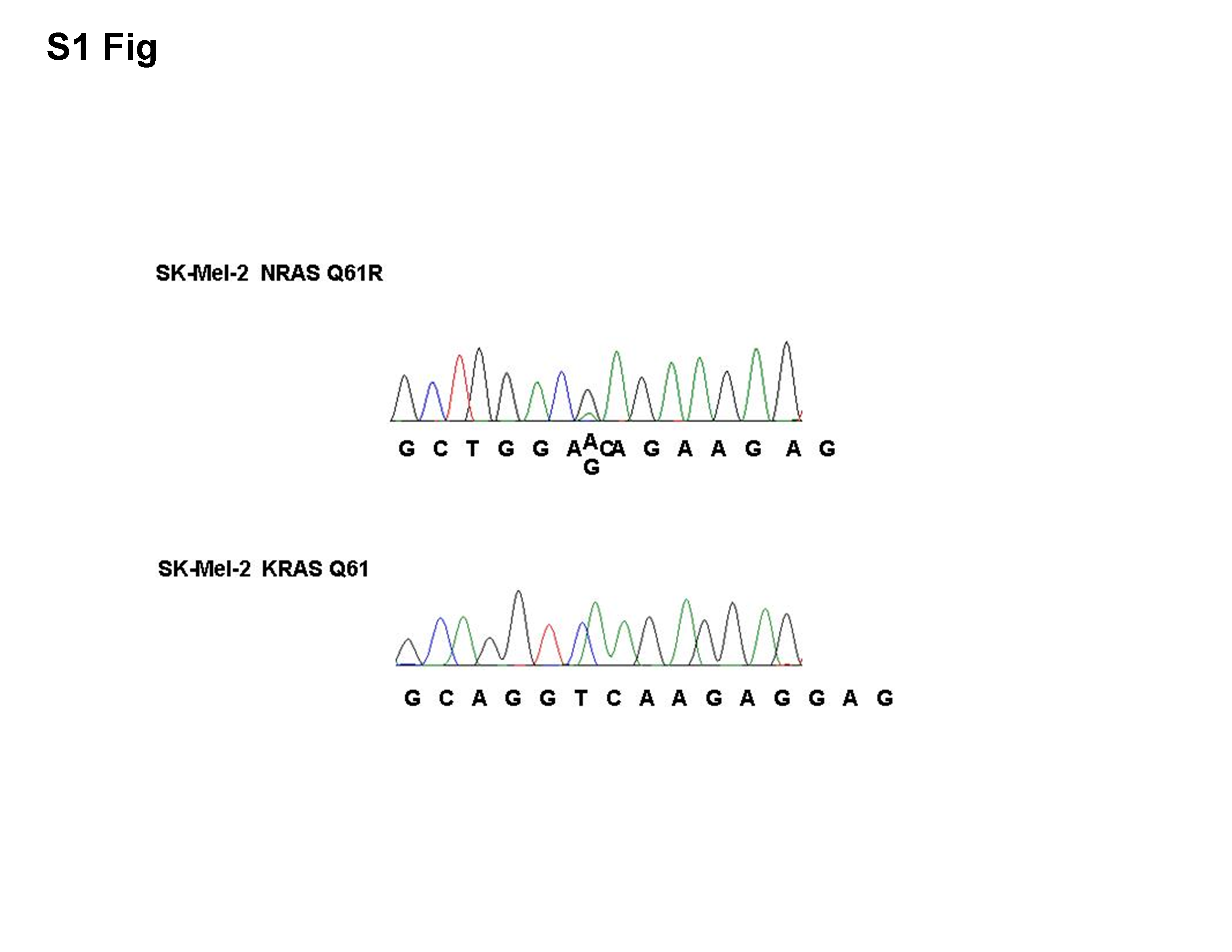

Supplement: S1 Fig — The human melanoma cell line SK-Mel-2 was screened for the NRASQ61R or KRASQ61R mutation. The sequencing of the amplified exon 3 of NRAS and KRAS in the SK-Mel-2 cells showed the mutation is NRASQ61R (top), and not KRASQ61R (bottom). (TIF) [file pone.0191264.s002.tif]
